# Circulating Nucleosomes and Histones in the Development of Lung Injury and Sepsis

**DOI:** 10.3390/cimb47020133

**Published:** 2025-02-19

**Authors:** Saugata Dutta, Sauradeep Dutta, Payaningal R. Somanath, S. Priya Narayanan, Xiaoyun Wang, Duo Zhang

**Affiliations:** 1 Clinical and Experimental Therapeutics, College of Pharmacy, University of Georgia and Charlie Norwood VA Medical Center, Augusta, GA 30912, USA; 2 Department of Computer Science & Engineering, Southern University Bangladesh, Chattogram 4210, Bangladesh

**Keywords:** nucleosome, histone, neutrophil, lung injury, sepsis, ARDS

## Abstract

Cellular nucleosomes—the structural and functional units of chromatin—are inherently present in cells. During cellular damage or cell death, nucleosomes are released into circulation, either actively or passively. Once released, nucleosomes can become immunogenic entities through various mechanisms. The nucleosomal proteins in nucleosomes, called histones, play a pivotal role in inducing immunogenicity. However, intact nucleosomes are more immunogenic than the histones alone, as nucleosomal double-stranded deoxyribonucleic acid (dsDNA) enhances its immunogenic potential. Our recent study has shown that circulating histones are predominantly nucleosomal histones rather than free histones. Consequently, circulating histones primarily function as integral parts of circulating nucleosomes rather than acting independently. Circulating nucleosomes and their associated histones are implicated in the pathogenesis of a wide array of diseases. Notably, they are critical in the pathogenesis of lung injury and sepsis. These diseases have high morbidity and mortality rates and lack early diagnostic biomarkers. Further investigation is required to fully elucidate the role of circulating nucleosomes and their associated histones in disease processes. This review aims to discuss the current understanding of circulating nucleosomes and histones in the pathogenesis of lung injury and sepsis, with a focus on the underlying mechanisms.

## 1. Circulating Nucleosomes and Histones

**Figure 1:**
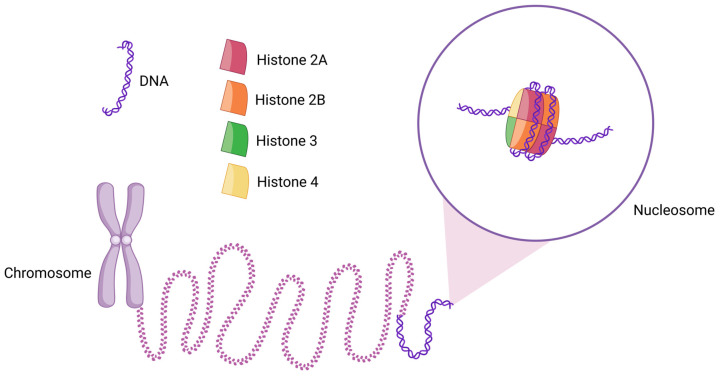
Schematic diagram of the structure of a nucleosome and its location in chromatin. Two copies of each histone H2A, H2B, H3, and H4 assemble to form the histone octamer, around which 145–147 bps (~1.7 turns) of DNA are wrapped. Thousands of nucleosomes collectively form chromatin, which appears as a “beads-on-a-string” structure. When further condensed, chromatin forms chromosomes.

A nucleosome is the basic structural [1,2,3,4] and functional unit of chromatin [5]. In a canonical nucleosome, a histone octamer is wrapped by 145–147 base pairs (bps) of DNA in a left-handed supercoil (Figure 1) [6]. The histone octamer is composed of two copies of each core histone protein—H2A, H2B, H3, and H4 [7]. Two H2A–H2B dimers and a (H3–H4) 2 tetramer form the histone octamer [8] with a two-fold symmetry axis [9]. The histone octamer binds and wraps ~1.7 turns of DNA. The addition of one histone H1 protein allows for the binding and wrapping of another 20 bps of DNA, resulting in two full turns around the octamer [10]. The lysine-rich histone H1 is present in almost all eukaryotic cells. However, in avian species and some other erythrocytes, histone H1 is mostly replaced by another lysine-rich histone, H5. This has become possible due to the transcriptional inertness of erythrocytes. Though many differences exist between the sequences of histones H1 and H5, these proteins have a very close structural similarity due to their three well-conserved domains—a basic N-terminal, an apolar central, and a basic C-terminal domain [11]. Thus, largely, histones H1 and H5 can be considered homologous.

Nucleosomes make it possible to package an enormously long genome inside a microscopic nucleus. For instance, in a human cell, 6 billion bps of DNA (~2 m of linear DNA) are accommodated within a 5–10 µm nucleus [10]. Forming the beads-on-a-string structure, up to 2 × 10^7^ nucleosomes can be compacted inside a minuscule nucleus [12]. In addition to their critical role in DNA packaging, nucleosomes are also crucially important for the regulation of nuclear processes—from gene expression to DNA replication, repair [13], recombination, and chromosome segregation [14]. Indeed, the role of nucleosomes is indispensable for the maintenance of cellular homeostasis and overall physiology. Nucleosomes can even pass on their epigenetic memory with high precision across mitotic generations. As a result, they can remember their position during DNA replication and RNA transcription [15].

**Figure 2:**
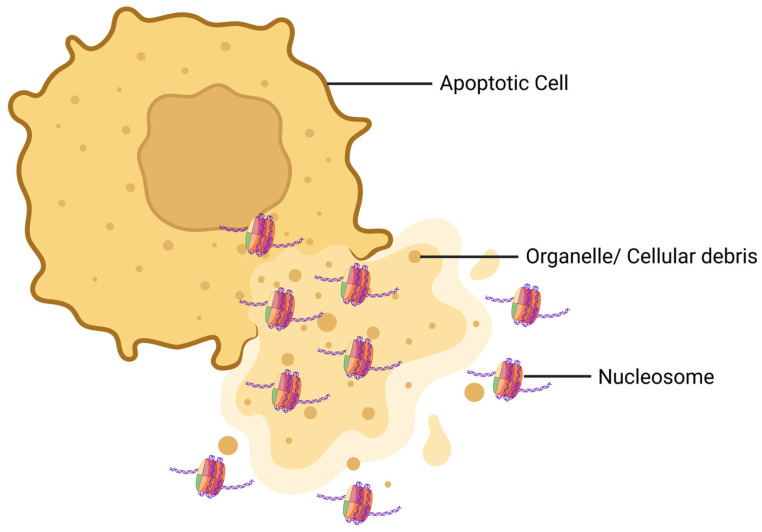
Schematic diagram of how nucleosomes are released from a dying cell. During apoptosis, the morphology of the cell is distorted and the cell membrane is ruptured. As a result, the nuclear and cytoplasmic contents of the dying cell are released. At this step, nucleosomes are released from the cell along with organelles and cellular debris.

Activated intracellular endonucleases cleave chromatin into mono- and oligo-nucleosomes [16]. Nucleosomes are released into the blood circulation from stressed and dying cells (Figure 2) [17]. As these nucleosomes are found in circulation, they are called circulating nucleosomes. The discharge of nucleosomes from cells can occur in both active and passive manners. Nucleosomes are actively secreted from cells through the amphisomes, whereas they are passively liberated from cells by the process of cell death [18]. Histone tails bulge from the octamer surface and undergo significant post-translational modifications (PTMs) [16]. Though studies indicate a strong association between circulating nucleosomes and inflammatory diseases [19,20], the specific role and the underlying mechanism of nucleosomes in lung injury and sepsis have not been thoroughly investigated.

## 2. Lung Injury and Sepsis

The alveolar epithelium can be damaged by various factors, including lung infections (caused by viruses, bacteria, or other pathogens), lung injury (resulting from the inhalation of gastric contents, toxic gases, or drowning), lung contusion, disseminated intravascular coagulation, sepsis, and severe pancreatitis. As a result, alveolar type 1 cells undergo injury or cell death, disrupting the alveolar–epithelial barrier. This disruption increases barrier permeability, promotes the accumulation of inflammatory cells, and impairs alveolar fluid clearance, ultimately leading to lung injury [21]. This condition is primarily referred to as acute lung injury (ALI) due to its rapid onset. Key clinical features of ALI include diffuse bilateral infiltrates and a reduced ratio of the partial pressure of arterial oxygen to the fraction of inspired oxygen (PaO_2_/FiO_2_). If hypoxemia becomes more severe, the condition progresses to acute respiratory distress syndrome (ARDS). During ALI and ARDS, the alveolar epithelium loses its functionality. The clinical impact of ALI and ARDS is very distressing. ALI and ARDS are associated with high incidence, morbidity, and mortality rates. The pathogenesis of ALI is highly complex, involving various factors that remain largely unexplored [22].

In contrast, chronic lung inflammation includes a pathological condition called chronic obstructive pulmonary disease (COPD). COPD patients experience the remodeling and inflammation of lower respiratory tracts and lung parenchyma, as well as the activation of immune and inflammatory processes [23]. COPD is considered the third leading cause of death worldwide [24]. The prevalence of COPD is increasing worldwide, primarily due to the increased consumption of cigarette smoking. Existing therapies are not very effective against COPD [23]. Another chronic lung injury syndrome is bronchopulmonary dysplasia (BPD) [25]. It is the leading cause of respiratory morbidity in preterm infants. BPD has a multifactorial and complex pathogenesis that involves various genetic and environmental factors [26]. The clinical phenotype of BPD is variable [27].

Sepsis is a severe and life-threatening condition resulting from an unregulated host response to infection, leading to organ dysfunction [28]. Sepsis can cause inflammatory damage across nearly all organ systems [29], with pneumonia being the most common cause of sepsis [28]. In sepsis, blood pressure drops, leading to decreased perfusion pressure and hypoxia. Globally, sepsis is one of the major causes of mortality, and its most severe form is called septic shock [30]. Septic shock is considered a distinct type of sepsis characterized by cellular, circulatory, and metabolic dysfunction, where the risk of mortality is extremely high [28]. Despite extensive research, the pathogenesis of sepsis remains poorly understood, particularly the immunopathogenesis. The highly heterogeneous nature of sepsis further complicates the development of effective therapeutic strategies [31].

## 3. Role of Nucleosomes/Histones in Pathology

Circulating histones can be found in circulation in two forms, i.e., DNA-bound histones (nucleosomes) and free histones. Nevertheless, previous studies have largely overlooked the distinction between nucleosomes and free histones. Some studies have even used the terms “nucleosomes” and “histones” interchangeably. As a result, the understanding of nucleosomes and free histones in circulation has remained unclear. Thus, this lack of clarity has hindered progress in subsequent studies. We distinguished and quantified the two forms of circulating histones in the circulation under physiological and pathological conditions. Our group has demonstrated, for the first time, that circulating histones are mostly present in the form of nucleosomes, rather than free histones, in the plasma of patients with COVID-19 [32]. Therefore, it is critically important to re-evaluate the contributions of these two different forms of histones to the immunopathology of disease processes [32].

Recent studies have shown that circulating nucleosomes are involved in the progression of various diseases and pathological processes [20,33]. Particularly, circulating nucleosomes can trigger significant inflammation, which can ultimately result in a cytokine storm [34]. Circulating nucleosomes exhibit stronger pro-inflammatory properties than circulating free (cf) histones and cfDNA [35]. Nucleosomes can contribute to inflammation in multisystem disorders such as chronic kidney disease [36]. A significantly higher concentration of circulating nucleosomes has constantly been observed in different pathological conditions such as sepsis [17], autoimmune diseases [37], stroke [38], degenerative diseases [39], and trauma [19].

One study demonstrated that among surviving trauma patients, those with higher levels of circulating histones were more likely to experience respiratory failure. The study showed a strong correlation between circulating histone concentrations and Sequential Organ Failure Assessment (SOFA) score, which is a scoring system used to evaluate the severity of multiple organ dysfunction. Additionally, the study found that sera from trauma patients with elevated histone levels were toxic to cells in vitro, and the toxic effects were reduced if the cells were treated with a synthetic antihistone. Similarly, sera from severe sepsis and pancreatitis patients also showed toxic effects on cells which were balanced after antihistone treatment. Elevated levels of circulating histones are highly evident in trauma, sepsis, and acid aspiration [40]. As these three pathological conditions can directly promote the pathogenesis of acute respiratory distress syndrome (ARDS) [41], circulating histones have a close association with ARDS. A study evidently found that circulating histones mediate ARDS pathogenesis [42]. Findings from another study showed that a slight increase in circulating histone levels may significantly worsen ALI [43]. Another recent study has found that inhibiting circulating histones prevents ALI in a murine model [44]. Plasma nucleosome levels of pediatric ARDS patients are associated with mortality, non-pulmonary organ failure, severity of ARDS [45], and decreased oxygenation [46].

Moreover, compared with healthy subjects, sepsis patients have an increased level of circulating nucleosomes [47], and this plasma level of circulating nucleosomes has an association with disease severity. Among sepsis patients, non-survivors have an elevated plasma level of nucleosomes compared to survivors [48]. In an ovine model, the neutralization of circulating histones can ameliorate septic shock [49]. Circulating histones induce endothelium injury, increased inflammatory response, and cascade coagulation activation, which ultimately results in sepsis [50]. Undoubtedly, histones are critically involved in the pathogenesis of sepsis [51] and septic shock [52]. Particularly, the level of circulating histone H3 is substantially increased during sepsis [53]. In children with meningococcal sepsis, circulating nucleosomes have a correlation with inflammatory response severity and an association with mortality [54].

**Figure 3:**
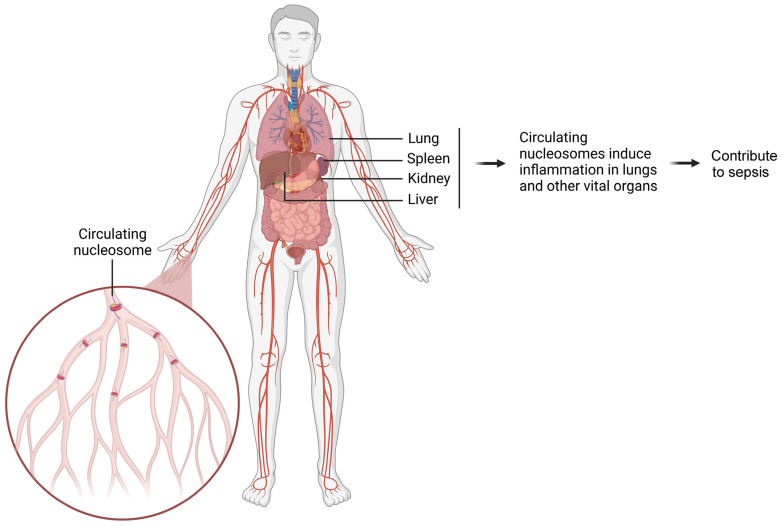
Schematic diagram illustrating how circulating nucleosomes promote inflammation in various organs and contribute to sepsis. The damaged organ releases a large number of nucleosomes into circulation. These circulating nucleosomes spread throughout the body and induce inflammation in vital organs. Ultimately, this cascade of inflammatory responses contributes to the development of sepsis.

Our study observed elevated levels of circulating nucleosomes in patients with ALI. As the first-line defense, macrophages immunologically respond to nucleosome exposure, which was demonstrated from nucleosome treatment on primary and immortalized macrophages of both human and murine origin. We also found that nucleosome-activated macrophages interact with lung epithelial cells. From a functional point of view, nucleosomes promote the activation of macrophages, as well as triggering local and systemic inflammation. The intratracheal administration of nucleosomes in murine lungs mimics local nucleosome release within the lung microenvironment during lung injury. Similarly, the intravenous administration of nucleosomes to murine models mimics the systemic distribution of nucleosomes to various organs during systemic inflammatory diseases and sepsis. These experiments have provided insights relating to the fact that nucleosomes can induce inflammation both locally and systemically, thereby exacerbating associated diseases. During inflammation, nucleosomes are released from the affected organ and disseminated throughout the body via circulation. This leads to the induction of further inflammation in the lungs and other vital organs, ultimately contributing to the development of sepsis (Figure 3). We have observed that during systemic inflammation, different organs, such as the lungs, kidneys, and liver, have shown evident signs of inflammation [5,32]. Furthermore, the systemic distribution of nucleosomes may mediate organ crosstalk [32,55], a process that maintains complex biological communication and feedback among individual organs through different mediators [55,56].

## 4. Contribution of Neutrophil–Histone Interplay in Pathology

**Figure 4:**
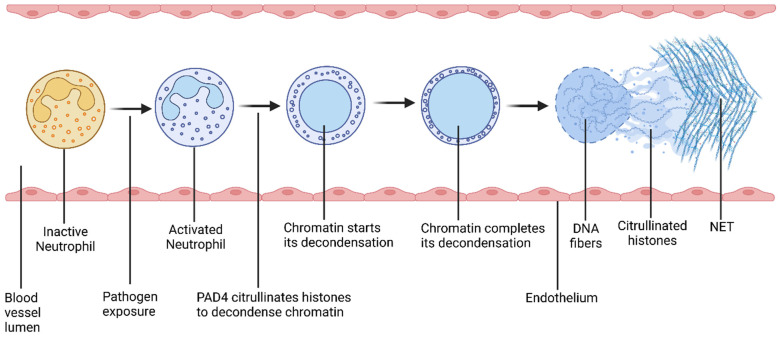
Schematic diagram of the process of NETosis. Pathogen exposure activates neutrophils. After activation, PAD4 citrullinates histones, which decondenses chromatin. As a result, neutrophil releases DNA fibers along with antimicrobial biomolecules including histones. These released DNA fibers form a web-like structure, called a neutrophil extracellular trap (NET). This process is called NETosis.

Neutrophils play a critical role in the development of histone-induced pathological conditions [57]. These cells are the most abundant type of white blood cells (WBCs), comprising up to 70% of circulating WBCs. In the presence of pathogens and/or inflammatory mediators, neutrophils promptly move to the area of infection or inflammation and become activated (Figure 4) [58]. Upon activation, histones undergo citrullination, a process where arginine is irreversibly converted into citrulline with the help of the peptidyl arginine deiminase (PAD) family of enzymes [59]. Notably, nuclear enzyme PAD4 hyper-citrullinates histones H2A, H3, and H4 [60]. Histone modifiers, including class I and IIb histone deacetylases, deacetylate histone H3 for citrullination via the PAD4 enzyme [61]. As a result of histone citrullination, chromatin is thoroughly decondensed and DNA fibers are released into circulation [60]. The DNA fibers are interwoven to form a web-like structure, called neutrophil extracellular traps (NETs) [62]. The process of NET formation is called NETosis [63].

NETs can directly [64] and indirectly mediate both innate and adaptive immune systems [65]. While NETs are a central part of the innate immune system [62,65,66,67], NETs can also substantially affect the adaptive immune system [68]. NETs can also mediate a connection between adaptive and innate immune responses [69,70]. However, as the NET response is non-specific, it can cause injury to alveoli, terminal bronchioles, the endothelium, and extracellular fibers, and may ultimately result in tissue damage [62]. NETs can cause immune dysregulation, which can lead to severe consequences [71,72]. For instance, dysregulated innate immune responses can cause systemic autoinflammatory disorders [73], whereas dysregulated adaptive immune responses can cause ALI and death [74]. Dysregulated NETosis can cause immune response modifications and result in inflammatory disorders [75].

NETs can effectively destroy various pathogens, such as viruses, bacteria, fungi, protozoa, etc. [76]. NETs can capture [62], inhibit the distribution and cellular uptake, and, ultimately, immobilize and inactivate various pathogens [60]. NETs can destroy pathogens due to the proteolytic and pro-inflammatory properties of NET-associated molecules [62]. DNA fibers of NETs convey different molecules that can promote either proteolysis or inflammation. These molecules include nuclear proteins and cytoplasmic proteins, including histones, elastase, cytokines, myeloperoxidase, pentraxin, and matrix metalloproteinases (MMPs), as well as bacterial peptides [62] and antimicrobial proteins [77]. Histones are particularly important among all these molecules. Initially, histones induce NETosis through citrullination. After the NETs are formed, histones act as antimicrobial peptides to directly kill a wide variety of pathogens. Thus, histones play a critical role both in the formation of NETs and the destruction of pathogens [78].

NET-associated histones can also promote the NET-related cell death of endothelial and epithelial cells [43]. A study showed that circulating histones collected from the plasma of severe trauma patients enhanced cell permeability and cytokine production in endothelial cells, as well as triggering NETosis in neutrophils. The study demonstrated that histone-induced NETosis can further promote endothelial damage. [79]. In the event of cellular injury that damages the epithelial or endothelial barriers of the vasculature, a feedback amplification loop is triggered. This allows cytokines, immune cells, and other damage-associated molecular patterns (DAMPs) to infiltrate the lungs, contributing to the development and progression of ARDS [79].

Margination is the process in which particles in the flowing blood migrate towards the wall of blood vessels. [80]. Due to margination, circulating neutrophils in the blood move towards the wall of blood vessels. This facilitates neutrophils to infiltrate alveoli. As the lungs are the largest site of neutrophil margination, neutrophil infiltration in the alveoli is a primary attribute of ARDS. The increased susceptibility of the lungs to circulating histone-induced injury is primarily attributed to histone-induced NETosis, as demonstrated in both preclinical [75] and clinical studies [81].

## 5. Contribution of Other Nucleosome-Related Factors in Pathology

Circulating histones may function as DAMP signals and can contribute to multiple organ failure syndrome. Studies have shown that circulating histones can cause endothelial cell dysfunction in vitro and in vivo. Recent research by Kim et al. (2022) demonstrated that histones H3 and H4 cause permeability and inflammation in endothelial cells. Moreover, histones H3 and H4 greatly augment pathogen-induced endothelial cell permeability and inflammation. In vivo, histone injection in lung injury mouse models exacerbates the condition by augmenting ALI parameters. A Toll-like receptor (TLR), called TLR4, but not TLR2 or TLR6, inhibits histone-induced endothelial cell dysfunction. Treatment with all histone proteins showed distinct cytotoxicity in both endothelial and epithelial cells. Various studies have demonstrated that histones contribute to sepsis and ARDS by impairing endothelial function [43]. However, it is unknown whether inflammatory mediators and DAMPs contribute to lung injury in critical care in the context of co-infections, such as mechanical ventilator-associated pneumonia in the COVID-19 pandemic [82].

Initial studies to understand the pathological importance of histones in different disease processes mostly focused on the function of histone deacetylase inhibitors. Recently, we have come to know that the circulating histones, particularly histones H3 and H4, are released into the blood in the event of sepsis. These circulating histones can result in the disruption of endothelial cells, organ failure, and death. According to a study, to induce inflammation, histones work as DAMP signals to interact with a pattern recognition receptor (PRR), called TLR9, and a gene, called myeloid differentiation primary response gene 88 (MyD88). TLR9 is activated in response to histone administration. TLR9- or MyD88-mutant mice do not respond to histone administration. This study proposed a ligand–receptor mechanism to justify histone-related cell damage. According to another study, in epithelial cells, the positively charged histones H1/H5 and H4 modify the transepithelial conductance due to their voltage-sensitive attributes [75].

The lung is particularly susceptible to histone-induced injury. Histones damage the lungs in a non-specific approach. Positively charged circulating histones bind to the negatively charged phospholipids of the endothelial cell membrane, inducing Ca^2+^ influx and disrupting the cell membrane that releases intracellular mediators. Additionally, circulating histones may induce coagulation disorders that can help in the development of ARDS. Elevated histone levels in the blood at the initial phase of trauma may cause trauma-induced early cytokine storms. In trauma patients, increased circulating histones can cause lung injury and multiple organ dysfunction [40].

The epigenetic modifications of histones significantly influence chromatin functions, leading to tissue differentiation and an array of changes in homeostasis. These modifications are associated with changes in fragmentation. The fragmentation of cfDNA is tightly regulated during homeostasis, with the resulting fragments closely linked to their tissues of origin and nucleosome structures. For example, the acetylation on lysine 27 of histone H3, as well as the trimethylation on lysine 4 of histone H3, shows various fragmentomic arrangements of cfDNA [83]. Since acetylation is the primary form of epigenetic modification in histones, histone deacetylases have shown that they can reduce the effects of histone-induced lung injury [84].

Moreover, nucleosomal dsDNA is also capable of inducing a significant level of immunogenic response. cGMP-AMP synthase is a pattern recognition receptor that can recognize DAMPs and pathogen-associated molecular patterns (PAMPs) by sensing cytoplasmic double-stranded DNA from different sources including chromatin. When dsDNA binds to cGMP-AMP synthase (cGAS), cGAS is activated to produce 2′, 5′- cyclic GMP-AMP (cGAMP), which is ultimately sensed by the stimulator of interferon genes (STING). The STING induces the synthesis of type-I interferons (such as IFN-α and IFN-β) and/or pro-inflammatory cytokines (such as IL-6, IL-8, TNF, etc.). In this way, nucleosomal dsDNA activates the cGAS-STING pathway and causes different pathophysiological events including inflammation-mediated cell senescence and carcinogenesis [20].

Mechanistically, our study has demonstrated that nucleosomes activate NF-κB signaling in order to cause inflammation, which can be reduced by inhibiting NF-κB signaling with Sulfasalazine. Our study has demonstrated that circulating nucleosomes are crucially important in the activation of macrophages and the inflammation of the lungs during ALI pathogenesis. In contrast to previous studies, our study utilized clinically rational doses of nucleosomes for both in vitro and in vivo experiments. It mimics the condition of acute lung inflammation and provides a clinically acceptable perspective regarding nucleosome-induced inflammation [32].

## 6. Diagnostic Potentials of Circulating Nucleosomes and Histones

Circulating nucleosomes play a critical role in the development of various diseases, making them valuable biomarkers for diagnosis [33,85]. By analyzing the plasma levels of circulating nucleosomes and their correlation with disease severity, they show great promise for early diagnosis and prognosis. For example, circulating nucleosomes are potential biomarkers for pediatric ARDS, helping predict disease severity and progression [79]. Study findings show that circulating histones can potentially act as biomarkers for ARDS [42,75]. Studies indicate that circulating histones, particularly H3 and H4, also serve as effective biomarkers and therapeutic targets in inflammatory diseases [86]. Their diagnostic potential has already been validated in a clinical setting in patients with systemic sclerosis-associated interstitial lung disease [87]. Circulating nucleosomes can also potentially be used as biomarkers for another inflammatory disease, i.e., severe acute pancreatitis [88], and can be considered as an early biomarker for lung injury [40].

In sepsis, circulating nucleosomes are emerging as promising biomarkers for both diagnosis and prognosis, correlating with disease severity and outcomes [19,35]. They may also predict fatal outcomes in septic shock patients [67]. Additionally, circulating nucleosomes, particularly histones H2B, H3, and H4, have been identified as key biomarkers in sepsis diagnosis and in predicting sepsis-induced mortality [74,75,76]. The lactylation of histone H3 (lysine 18) may indicate the severity of advanced sepsis [77], and citrullinated histone H3 shows potential as a diagnostic and prognostic biomarker for conditions like sepsis, septic acute pancreatitis, and sepsis-associated encephalopathy [78,79,80,81].

## 7. Conclusions

Lung injury and sepsis are among the dreadful diseases that have high rates of morbidity and mortality, and existing therapeutics and diagnostics for these diseases are not very effective. Circulating nucleosome/histone-induced immunogenicity is crucially involved in disease processes, particularly lung injury and sepsis. The interaction between neutrophils and histones plays a crucial role in this pathogenesis. Additionally, other nucleosome-based mechanisms are also being explored in relation to pathogenesis. Nucleosomes and histones have a high potential as diagnostic biomarkers.

One notable limitation is that the majority of the current experimental findings are derived from in vitro and preclinical studies, making it essential to validate these findings in clinical settings to assess their feasibility and relevance. It can be challenging to replicate all the experiments in a clinical setting. Some challenges may not be overcome in a preclinical setting. For instance, due to the critical role of histones in the maintenance and overall function of cells, it is not feasible to create a nucleosome/histone-deficient animal model knockout due to embryonic lethality. As a result, this limitation has hindered the ability to conduct “loss-of-function” studies, which are valuable tools for assessing their pathological roles.

In addition, circulating nucleosomes and their associated histones are released into circulation during cellular stress. Nevertheless, the molecular mechanism of pathogenesis is still unclear. Since our current understanding is not adequate to address the nucleosome/histone-induced pathogenesis of lung injury and sepsis, a thorough and multi-dimensional investigation is required in this context. To ensure the ultimate success of such research and related efforts, it is crucial to translate the experimental findings into clinical applications. This will require both preclinical and clinical studies to facilitate this translation.
